# Recent advances in pancreatology

**DOI:** 10.3389/fphys.2014.00300

**Published:** 2014-08-11

**Authors:** Atsushi Masamune

**Affiliations:** Division of Gastroenterology, Tohoku University Graduate School of MedicineSendai, Japan

**Keywords:** autoimmune pancreatitis, cystic fibrosis transmembrane conductance regulator, epithelial-mesenchymal transition, fibrosis, pancreatic cancer, pancreatic stellate cells, pancreatitis, trypsin

Pancreatic diseases, including acute and chronic pancreatitis (CP) and pancreatic cancer, are intractable. In recent years, great advances have been made in the field of pancreatology: the pathogenesis, diagnostic modalities, and development of novel therapeutic interventions. This E-Book is derived from the *Frontiers in Physiology section Gastrointestinal Sciences* Research Topic entitled “Recent Advances in Pancreatology.” Its goal is to bring established experts to present state-of-art studies in pancreatic physiology, and to provide ideas on different approaches useful to research challenges. This book presents nine contributions, in the form of reviews, hypothesis and theory article, and original article.

The articles can be mainly classified into three categories: pancreatitis, autoimmune pancreatitis (AIP), and pancreatic cancer. To date, several pancreatitis-associated genes have been identified such as the cationic trypsinogen (*PRSS1*) gene and the serine protease inhibitor, Kazal type 1 (*SPINK1*) gene have been identified. The review article by Whitcomb (2012), who had originally identified the mutations in the *PRSS1* gene as a cause of hereditary pancreatitis, presented a new framework for the interpretation of genetic variants in patients with CP based on modeling and simulation of physiological processes with or without genetic, metabolic, and environmental variables. This framework is especially important when we deal with billions of sequencing data obtained by the next-generation sequencers. Ohmuraya et al. (2012) reviewed the old and new roles of the intrapancreatic *SPINK1/Spink3* expression in the development of pancreatitis. In addition to the established roles as a trypsin inhibitor, SPINK1 is involved in autophagy, cell growth, and cell death in pancreatic acinar cells and cancer cells. The precise molecular mechanisms of intraductal pancreatic stone formation in CP are largely unknown. Ko et al. (2012) reported that the mislocalization of the cystic fibrosis transmembrane conductance regulator (CFTR) is a cause of protein plug formation, leading to the formation of pancreatic stones in CP. CFTR was largely mislocalized to the cytoplasm of pancreatic duct cells in CP, including AIP. Because corticosteroids normalized the localization of CFTR to the proper atypical membrane, Ko et al. concluded that corticosteroids might be useful to prevent protein plug and stone formation in patients with CP.

Pancreatic stellate cells (PSCs) have attracted increasing attention from researchers. Apte et al. (2012), who originally identified PSCs in rats, reviewed the current knowledge about the roles of PSCs in normal and diseased pancreas. In healthy pancreas, PSCs may maintain normal tissue architecture and act as progenitor cells, immune cells, and an intermediary in exocrine secretion in the pancreas. It has been established that PSCs play a critical role in pancreatic fibrosis, a consistent histological feature of CP and pancreatic cancer. PSCs interact closely with pancreatic cancer cells facilitating cancer progression. Several therapeutic strategies targeting PSCs have been examined in experimental models of CP and pancreatic cancer, although their clinical usefulness remains a challenge.

AIP has been increasingly recognized as a distinctive type of pancreatitis with a presumed autoimmune etiology. The molecular mechanisms responsible for the development of AIP are largely unknown. As reviewed by Haruta et al. (2012), the induction of AIP-like pancreatic lesions by viral and bacterial components in mice suggests a role of commensal flora in the development of AIP. From the clinical point of view, Kamisawa et al. (2012) gives an overview of AIP including its concept, the international consensus diagnostic criteria (ICDC) and standard therapeutic regimen. The goals of the ICDC for AIP are to develop criteria that can be applied worldwide, taking marked differences in practice patterns into consideration, to safely diagnose AIP and avoid misdiagnosis of pancreatic cancer as AIP. According to the ICDC, AIP has been classified into two subtypes: type 1 related with IgG4 (lymphoplasmacytic sclerosing pancreatitis) and type 2 with granulocytic epithelial lesion (idiopathic duct-centric CP). The ICDC would contribute to further clarification of the clinical features, pathogenesis, and natural history of AIP around the world.

Lastly, three articles focus on pancreatic cancer. The epithelial-mesenchymal transition (EMT) is a developmental process that allows a polarized epithelial cell to undergo multiple biochemical changes that enable it to assume a mesenchymal phenotype. The phenotype associated with EMT includes enhanced migratory capacity, invasiveness, elevated resistance to apoptosis, and greatly increased production of extracellular matrix components. Thus, EMT plays a critical role in cancer progression. Hamada et al. (2012) reviewed the regulators of EMT in pancreatic cancer. In addition to multiple cytokines, growth factors and downstream transcriptional factors, non-coding RNA including microRNA contributes to EMT. Satoh et al. (2012) focus on MSX2, a member of the homeobox genes family, as an inducer of EMT in pancreatic cancer. MSX2 enhances the malignant phenotypes of pancreatic cancer, and evaluating MSX2 levels might be useful to differentiate pancreatic cancer from CP. Mizuno et al. (2013) describe that leucine-rich-repeat-containing G-protein-coupled receptor 5 (LRG5), a marker of intestinal stem cells, was expressed in the cytoplasm of pancreatic cancer cells. LRG5 was not co-localized with CD133, a cancer stem cell marker, in either neoplastic or non-neoplastic tissues. Further studies are required whether LRG5 expression is useful as an indicator of the prognosis.

In summary, the articles in this E-book will contribute to deepening our knowledge in both basic and clinical research in the field of pancreatology. Further understanding will underpin rational approaches to the treatment of intractable pancreatic diseases.

## Conflict of interest statement

The author declares that the research was conducted in the absence of any commercial or financial relationships that could be construed as a potential conflict of interest.
